# Relevance of pepsinogen, gastrin, and endoscopic atrophy in the diagnosis of autoimmune gastritis

**DOI:** 10.1038/s41598-022-07947-1

**Published:** 2022-03-10

**Authors:** Hiroshi Kishikawa, Kenji Nakamura, Keisuke Ojiro, Tadashi Katayama, Kyoko Arahata, Sakiko Takarabe, Aya Sasaki, Soichiro Miura, Yukie Hayashi, Hitomi Hoshi, Takanori Kanai, Jiro Nishida

**Affiliations:** 1 Department of Gastroenterology, Ichikawa General Hospital, Tokyo Dental College, 5-11-13 Sugano, Ichikawa, Chiba 272-8513 Japan; 2 Department of Pathology and Laboratory Medicine, Ichikawa General Hospital, Tokyo Dental College, Ichikawa, Chiba Japan; 3 Graduate School, International University of Health and Welfare, Minato-ku, Tokyo, Japan; 4 Center for Diagnostic and Therapeutic Endoscopy, Keio University Hospital, Tokyo, Japan; 5 Division of Gastroenterology and Hepatology, Department of Internal Medicine, Keio University, Shinjuku-ku, Tokyo, Japan

**Keywords:** Gastritis, Diagnostic markers, Autoimmunity, Gastrointestinal hormones, Oesophagogastroscopy

## Abstract

Simple objective modalities are required for evaluating suspected autoimmune gastritis (AIG). This cross-sectional study aimed to examine whether pepsinogen, gastrin, and endoscopic findings can predict AIG. The diagnostic performance of endoscopic findings and serology in distinguishing AIG was evaluated. AIG was diagnosed in patients (N = 31) with anti-parietal cell antibody and/or intrinsic factor antibody positivity and histological findings consistent with AIG. Non-AIG patients (N = 301) were seronegative for anti-parietal cell antibodies. Receiver operating characteristic curve analysis of the entire cohort (N = 332) identified an endoscopic atrophic grade cutoff point of O3 on the Kimura–Takemoto classification (area under the curve [AUC]: 0.909), while those of pepsinogen-I, I/II ratio, and gastrin were 20.1 ng/mL (AUC: 0.932), 1.8 (AUC: 0.913), and 355 pg/mL (AUC: 0.912), respectively. In severe atrophy cases (≥ O3, N = 58, AIG/control; 27/31), the cutoff values of pepsinogen-I, I/II ratio, and gastrin were 9.8 ng/mL (AUC: 0.895), 1.8 (AUC: 0.86), and 355 pg/mL (AUC: 0.897), respectively. In conclusion, endoscopic atrophy is a predictor of AIG. High serum gastrin and low pepsinogen-I and I/II ratio are predictors even in the case of severe atrophy, suggesting their usefulness when the diagnosis of AIG is difficult or as serological screening tests.

## Introduction

Autoimmune gastritis (AIG) is an uncommon chronic gastritis characterized by immune-mediated destruction of parietal cells^1,2^. AIG develops by the following two mechanisms: (1) a decrease in parietal cells resulting in hypochlorhydria or achlorhydria, and a lack of intrinsic factor; and (2) corpus-restricted inflammation progressing to severe oxyntic gland atrophy^2,3^. Hypergastrinemia, neuroendocrine tumor, and iron deficiency anemia induced by hypochlorhydria; macrocytic-megaloblastic anemia induced by intrinsic factor-dependent vitamin B-12 deficiency; and low serum pepsinogen (PG) I secondary to advanced oxyntic gastric atrophy are the major clinical findings. Although it is clinically important to detect AIG considering the high coincidence rates of gastric cancer, neuroendocrine tumors, macrocytic-megaloblastic anemia, and other autoimmune disorders^4^, diagnosis is usually difficult, and most cases are overlooked because of their nonspecific and subtle clinical manifestations^5,6^.

The two diagnostic autoantibodies of this disease are the anti-parietal cell antibody (APCA) and anti-intrinsic factor antibody (AIFA)^3^; however, AIG cannot be diagnosed merely based on these autoantibodies, and they are not recommended for screening owing to lack of cost-effectiveness, the high false positive rate of APCA, and low sensitivity of AIFA^7,8^. Histological examination is another established diagnostic tool for AIG^2,3^; however, despite its high sensitivity, negative results cannot always rule out AIG because of the inherent limitations of biopsy in only a limited area of the gastric mucosa. Since distinct diagnostic criteria for AIG have not yet been established, the method of diagnosis differs considerably among investigators and includes histology alone^9,10^; histology and autoantibodies^11–13^; histology, autoantibodies, endoscopy, and gastrin^14^; histology and serology^15^; autoantibodies and endoscopy^16^; autoantibodies or pernicious anemia; and endoscopy and serology without any histological information^17^. However, the most reliable and objective finding would be a combination of autoantibodies and histology, which is considered the gold standard by several investigators^18^. Thus, we aimed to examine other potential predictors of AIG, focusing on endoscopic findings, PG, and gastrin.

PG and gastrin are useful in establishing a diagnosis of AIG when autoantibody and histological findings are inconclusive, for example, when false positive or false negative results are observed in strongly suspected cases. Furthermore, these markers are included in the serological screening tests such as the ABC method (PG)^19^ and Gastropanel® (PG and gastrin)^20^. Using these tests, clinicians can effectively evaluate the presence of suspected AIG without any histological evidence.

Macroscopic endoscopic findings are also clinically significant as the potential first diagnostic clues of AIG. Although the most common initial findings of AIG are hematological disorders^12,21^, several investigators suggested that gastrointestinal symptoms are another important clue. Carabotti et al. reported that 56.7% of AIG cases are associated with gastrointestinal symptoms, 69.8% of which are dyspepsia^22^. Soykan et al. reported that an unexpectedly high rate of 50.4% of patients with AIG complained of abdominal symptoms^23^. Miceli et al. reported that 34% of AIG cases were diagnosed according to histological findings of gastritis after endoscopy^12^, and a multicenter study in Japan demonstrated that the most common modality for diagnosing AIG was endoscopy^17^. These studies suggest the importance of endoscopy as an initial detection tool in some patients with AIG who have abdominal symptoms or those who are negative for anemia or other autoimmune diseases.

In this cross-sectional study, we assessed the performance of endoscopic findings and serology (PG and gastrin) in diagnosing AIG in (1) patients with AIG strictly defined based on autoantibody positivity and characteristic histological findings, and (2) non-AIG patients seronegative for autoantibodies.

## Results

### Baseline characteristics of the whole study population

**Figure 1:**
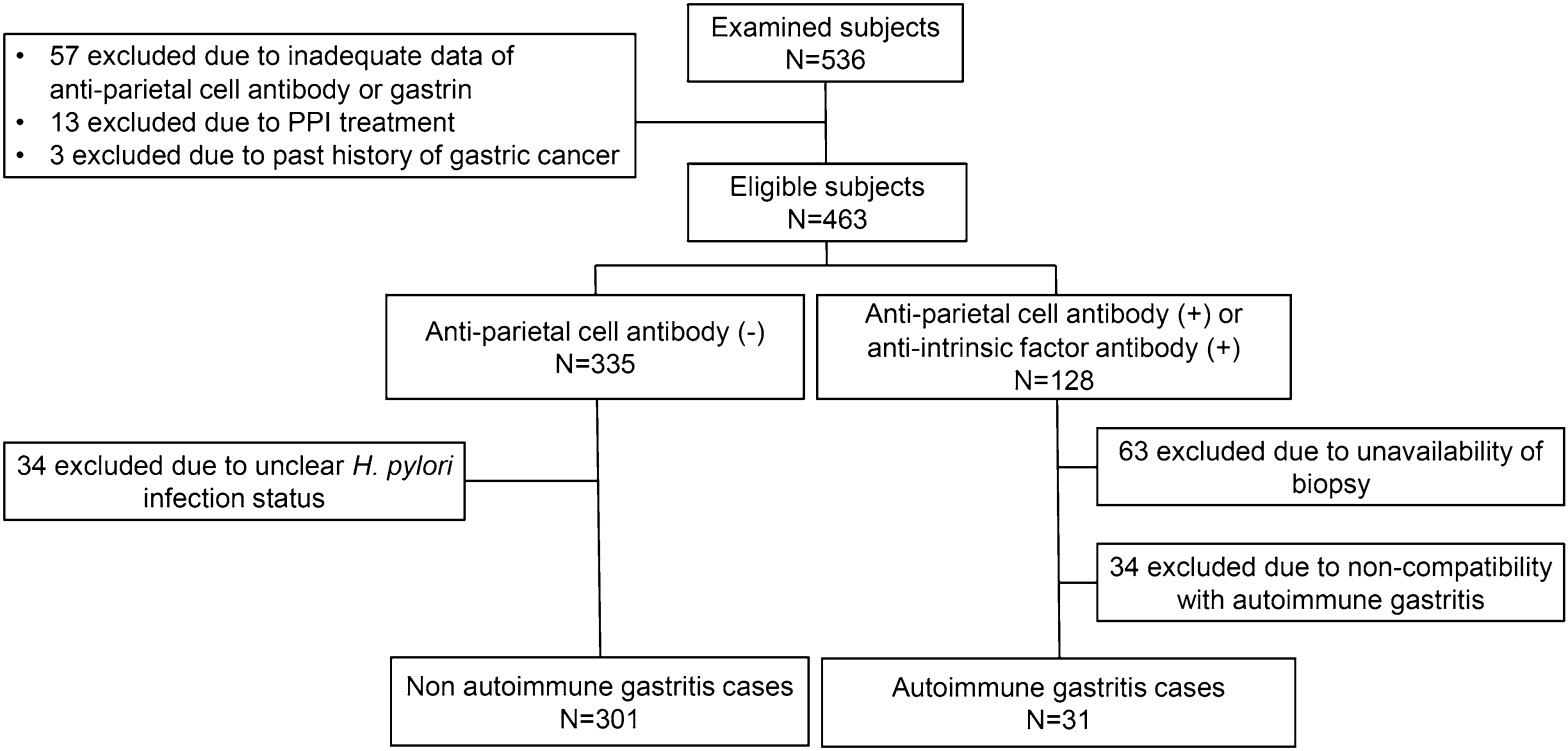
Study flowchart of patient selection.PPI, proton pump inhibitor.

As shown in Fig. 1, 301 non-AIG patients (male, 53.8%; median age [interquartile range (IQR)], 70 [63.5–75] years) and 31 patients with AIG (male, 58.1%; median age [IQR], 73 [66–76] years) were finally enrolled for analysis in this study. Among patients with AIG, 13 initially presented to our hospital, and the remaining 18 were already being followed up at our hospital. The reasons for performing endoscopy were as follows: positive results in the ABC method (n = 14, 45.2%), abnormalities on barium X-ray (n = 5, 16.1%), annual endoscopic follow-up (n = 3, 9.7%), screening in asymptomatic individuals (n = 6, 19.3%), and others (n = 3, 9.7%).

Age, sex, serum biomarker levels (PG and gastrin), *Helicobacter pylori* (*H. pylori*) serology, and autoantibodies were evaluated for all patients, as shown in Table 1. Supplementary Table S1 shows the detailed histological findings in patients with AIG (according to our definition). Oxyntic mucosal atrophy and lymphocyte and plasma cell infiltration were observed in all cases. Supplementary Table S2 presents the observed Kappa values among the three endoscopists. As a Kappa value of ≥ 0.60 is usually considered reliable and applicable for evaluation, the “endoscopic atrophic border” (mean, 0.654) and disappearance of the gastric fold (mean, 0.622) were adapted as parameters to be analyzed in this study. However, three other endoscopic findings, including corpus-predominant advanced atrophy compared to the antral part (mean, 0.335), sticky mucosa (mean, 0.352), and hyperplastic polyp (mean, 0.516), were excluded from the analysis as endoscopic findings generally are not highly reliable.

**Table 1:** Baseline characteristics of all participants.

Baseline characteristics	Autoimmune gastritis (N = 31)	Non-autoimmune gastritis (N = 301)	*P*-value*
Male, N (%)	18 (58.1)	162 (53.8)	0.652
Age, years, median (IQR)	73 (66–76)	70 (63–75)	0.132
Gastric cancer, N (%)	2/31 (6.5%)	8/301 (2.7%)	0.237
Pepsinogen I (ng/mL), median (IQR)	6.4 (3.8–15.9)	43.3 (31.2–59.5)	< 0.001*
Pepsinogen II (ng/mL), median (IQR)	7.7 (4.9–10.3)	8.3 (5.6–13.0)	0.273
Pepsinogen I/II ratio, median (IQR)	1.0 (0.6–1.5)	5.5 (3.6–7.2)	< 0.001*
*H. pylori* antibody titer > 10 U/mL, N (%)	4/31 (12.9%)	105/301 (34.9%)	< 0.05*
*H. pylori* infection status Uninfected/present infection/eradicated/past infection, N (%)	17/6/8/0 (54.9%/19.4%/25.8%/0%)	77/106/89/29 (25.6%/35.2%/29.6%/9.6%)	< 0.01*
Gastrin (pg/mL), median (IQR)	1,310 (448–2,490)	102.0 (82–144)	< 0.001*
< 100/100–350/350–1,000/ > 1,000 pg/mL, N (%)	2/3/7/19 (6.5%/9.7%/22.6%/61.3%)	141/140/15/5 (46.8%/46.5%/5.0%/1.7%)	< 0.001*
Anti-parietal cell antibody positive, N (%)	29/31 (93.5%)	0/301 (0%)	< 0.001*
Anti-intrinsic factor antibody positive, N (%)	15/27 (55.6%)	NA	NA
Endoscopic atrophic border^†^, N (%) C0/C1–3/O1–2/O3/O4, N	0/2/2/7/20 (0%/6.5%/6.5%/22.6%/64.5%)	71/67/132/22/9 (23.5%/22.3%/43.9%/7.3%/3.0%)	< 0.001*
Endoscopic atrophic border^†^ > O3, N (%)	27/31 (87.1%)	31/301 (10.2%)	< 0.001*
Disappearance of the gastric fold, N (%)	19/31 (61.2%)	20/301 (6.6%**)**	< 0.001*

Data are provided as numbers (%) or median (interquartile range [IQR]).

*P-value: Fisher’s exact test or Man–Whitney *U* test; autoimmune gastritis vs. non-autoimmune gastritis.

^†^Endoscopic atrophic border was based on the Kimura–Takemoto classification. We defined O4 as marked vascular visibility observed in the greater curvature of the corpus, which is defined as O3 in the Kimura–Takemoto classification.

The clinical characteristics of patients with AIG and non-AIG gastritis were compared (Table 1). The median PG I levels (6.4 [3.8–15.9] vs. 43.3 [31.2–59.5] ng/mL, *P* < 0.001; normal value, < 70 ng/mL) and PG I/II ratios (1.0 [0.6–1.5] vs. 5.5 [3.6–7.2], *P* < 0.001; normal value, < 3) were significantly lower, and gastrin levels (1,310 [448–2,490] vs. 102.0 [82–144] pg/mL, *P* < 0.001; normal value, < 140 pg/mL) were significantly higher in patients with AIG than in those without. Severe endoscopic atrophy (atrophic border of O3 and O4) (87.1% vs. 10.2%, *P* < 0.001) and the disappearance of the gastric fold (61.2% vs. 6.6%, *P* < 0.001) were significantly more common in patients with AIG than in those without. Age, sex, PG II level, and the prevalence of gastric cancer were similar between the two groups. The detailed clinical findings of AIG are shown in Table 2. Briefly, urea breath tests (≥ 5%) and *H. pylori* serology (≥ 10 U/mL) were positive in 8/20 (40%) and 4/31 (12.9%) patients, respectively. Two-thirds of patients with AIG (20/31, 64.6%) showed a moderate to high titer (≥ 20) of APCA. Anemia, pernicious anemia, and Hashimoto’s thyroiditis were diagnosed in 8/31 (25.8%), 6/23 (26.1%), and 11/27 patients (40.7%), respectively. One case each of primary biliary cholangitis and Hunter’s glossitis was diagnosed before AIG diagnosis.

**Table 2:** Detailed clinical and laboratory findings of autoimmune gastritis.

Baseline characteristics	Autoimmune gastrits (N-31)
Diagnosis of *H. pylori* infection	
Urea breath test*	
> 5‰/2.5–5‰/ < 2.5‰, N (%)	8/6/6 (40%/30%/30%)
*H. pylori* antibody titer	
≥ 10 U/mL /3–9.9 U/mL / < 3 U/mL, N (%)	4/7/20 (12.9%/22.6%/64.5%)
***H. pylori* culture†**	
positive, N (%)	2/12 (16.7%)
**Anti-parietal cell antibody titer**	
Serum dilution (negative/1:10/1:20–40/1: ≥ 80), N (%)	2/9/14/6 (6.5%/29%/45.2%/19.4%)
**Associated disorders**	
Anemia, N (%)^‡^	8/31 (25.8%)
Low Vitamin B 12, N (%)^§^	12/23 (52.2%)
Pernicious anemia, N (%)^§^	6/23 (26.1%)
Hashimoto's disease, N (%)^||^	11/27 (40.7%)
**Gastric cancer, N (%)**	2/31 (6.5%)
intestinal/diffuse, N	1/1
early/advanced, N	2/0

*The urea breath test was performed in 20 participants.

^†^*H. pylori* culture was performed in 12 participants.

^‡^Anemia was defined as hemoglobin < 13.0 g/dL in men or < 11.4 g/dL in women.

^§^Vitamin B12 and pernicious anemia were evaluated in 23 participants. Low vitamin B12 was defined as vitamin B12 level < 233 pg/mL. Pernicious anemia was defined as vitamin B12 < 233 pg/mL, mean corpuscular volume > 80 fl, and hemoglobin < 13 g/dL in men or 11.4 g/dL in women.

^||^Hashimoto’s disease was evaluated in 27 participants.

### Diagnostic ability of each clinical parameter in predicting AIG

**Figure 2:**
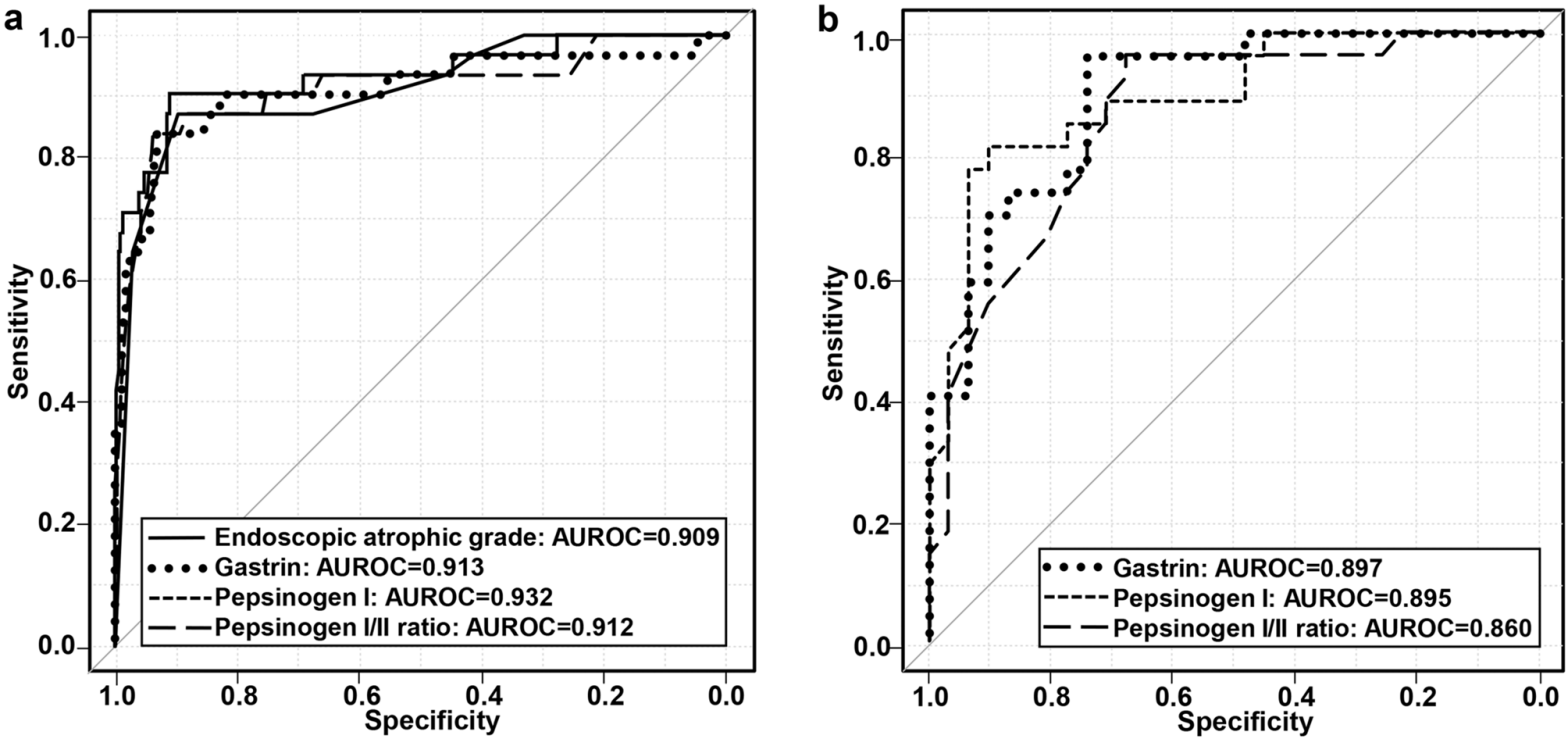
(**A**) Receiver operating characteristic curves showing the ability of each variable to distinguish autoimmune gastritis in the overall cohort. Receiver operating characteristic curves for the performance of endoscopic atrophy, disappearance of the gastric fold, pepsinogen I level, pepsinogen I/II ratio, and gastrin in distinguishing patients with autoimmune gastritis (N = 31) from those with non-autoimmune gastritis (N = 301). Area under the receiver operating characteristic curve (AUROC) of endoscopic atrophic grade, the disappearance of the gastric fold, gastrin, pepsinogen I, and pepsinogen I/II ratio was 0.909, 0.773, 0.913, 0.932 and 0.912, respectively; additionally, the optimal cutoff points for endoscopic atrophic grade, gastrin, pepsinogen I, and pepsinogen I/II ratio were, O3, 355 pg/mL, 20.1 ng/mL, and 1.8, respectively. (**B**) Receiver operating characteristic curves showing each variable’s ability to distinguish autoimmune gastritis in patients with severe endoscopic atrophy (≥ O3 on the Kimura–Takemoto classification). Receiver operating characteristic curves for the performance of pepsinogen I level, pepsinogen I/II ratio, and gastrin in distinguishing patients with autoimmune gastritis (N = 27) from those with non-autoimmune gastritis (N = 31). Area under the receiver operating characteristic curve (AUROC) of gastrin, pepsinogen I, and pepsinogen I/II ratio was 0.897, 0.895 and 0.86, respectively, and their optimal cutoff values were 355 pg/mL, 9.8 ng/mL, and 1.8, respectively.

The diagnostic ability of endoscopic findings, gastrin, and PG for predicting AIG was evaluated by ROC analysis of the entire study cohort (N = 332, AIG = 31, control = 301) (Table 3). The AUROC for an endoscopic atrophic border was 0.909 (95% CI 0.848–0.970), and the threshold point was O3 on the Kimura–Takemoto classification, with a sensitivity of 87.1%, specificity of 89.7%, and overall accuracy of 89.5%. The AUROC for the disappearance of the gastric fold was 0.773 (95% CI 0.66–0.879) with a sensitivity of 61.3%, specificity of 93.4%, and overall accuracy of 90.4%. The optimal cutoff value of gastrin was 355 pg/mL (AUROC, 0.912; 95% CI 0.844–0.983), with a sensitivity of 83.9%, specificity of 93.4%, and overall accuracy of 92.5%, while that of PG I was 20.1 ng/mL (AUROC, 0.932; 95% CI 0.874–0.990), with a sensitivity of 90.3%, specificity of 91.0%, and overall accuracy of 91.0%. The optimal cutoff value of the PG I/II ratio was 1.8 (AUROC, 0.913; 95% CI 0.840–0.981), with a sensitivity of 83.9%, specificity of 93.7%, and overall accuracy of 92.8%. The difference in the AUROCs of the endoscopic atrophic border and disappearance of the gastric fold was significant *(P* < 0.01); however, those between the endoscopic atrophic border and gastrin, PG I, and I/II ratio were not significant, according to the Delong test. Figure 2A illustrates the comparison of AUROCs for the endoscopic atrophic grade, PG I, PG I/II ratio, and gastrin, showing that all AUROCs were ≥ 0.80.

**Table 3:** Diagnostic performance of clinical parameters in predicting autoimmune gastritis in the overall cohort.

Clinical parameters	AUROC	SE	95% CI	Cutoff value	Sensitivity (%)	Specificity (%)	PPV (%)	NPV (%)	Accuracy (%)
Endoscopic atrophic grade*	0.909	0.031	0.848–0.970	O3	87.1	89.7	46.6	98.5	89.5
Disappearance of the gastric fold†	0.773	0.054	0.66–0.879	Disappearance of the gastric fold	61.3	93.4	48.7	95.9	90.4
Serum gastrin concentration (pg/mL)	0.913	0.035	0.844–0.981	355	83.9	93.4	56.5	98.3	92.5
Pepsinogen I (ng/mL)	0.932	0.03	0.874–0.990	20.1	90.3	91	50	98.5	91
Pepsinogen I/II ratio	0.912	0.03	0.840–0.983	1.8	83.9	93.7	58.1	97.9	92.8

*AUROC* area under the receiver operating characteristic curve, *95% CI* 95% confidence interval, *NPV* negative predictive value, *PPV* positive predictive value, *SE* standard error.

*Endoscopic atrophic grade was based on the Kimura–Takemoto classification (C0–O3), and O4 is defined as marked vascular visibility observed in the greater curvature of the corpus.

^†^*P* < 0.05 in comparison with the AUROC for the disappearance of the gastric fold, by the Delong test.

### Diagnostic ability of each serological parameter in predicting AIG in patients with advanced atrophy

The subgroup of patients with severe endoscopic atrophy ≥ O3 (N = 58 [AIG = 27, control = 31]) was extracted (Supplementary Table S3), and the diagnostic ability of gastrin and PG in predicting AIG in this subgroup of patients was also evaluated by ROC analysis. The median values of PG I (5.9 ng/mL vs. 18.9 ng/mL, *P* < 0.001; normal value, < 70 ng/mL), PG I/II ratio (0.9 vs. 3.3, *P* < 0.001; normal value, < 3), and prevalence of *H. pylori* seropositive cases (7.4% vs. 54.8%, *P* < 0.001; normal value, < 140 pg/mL) were significantly lower and the median gastrin level (1310 pg/mL vs. 102 pg/mL, *P* < 0.001) was significantly higher in patients with AIG than in those without. The prevalence of severe endoscopic atrophy extending to the entire corpus (O4) was significantly higher in patients with AIG than in those without (80.6% vs. 10.7%, *P* < 0.001). ROC analysis revealed that the optimal cutoff value of gastrin was 355 pg/mL (AUROC, 0.897; 95% CI 0.819–0.975), with a sensitivity of 96.3%, specificity of 74.2%, and overall accuracy of 84.5%, while that of PG I was 9.8 ng/mL (AUROC, 0.895; 95% CI 0.813–0.977), with a sensitivity of 81.5%, specificity of 90.3%, and overall accuracy of 86.2%. The optimal cutoff value of the PG I/II ratio was 1.8 (AUROC, 0.86; 95% CI 0.765–0.956), with a sensitivity of 96.3%, specificity of 67.7%, and overall accuracy of 81.0%. The AUROCs of PG I and I/II ratio were not significantly different from that of serum gastrin according to the Delong test (Table 4). Figure 2B shows a comparison of AUROCs between PG I, PG I/II ratio, and gastrin.

**Table 4:** Diagnostic performance of serum gastrin and pepsinogen in predicting autoimmune gastritis in cases with severe endoscopic atrophy (≥ O3 on the Kimura–Takemoto classification).

Clinical parameters	AUROC	SE	95% CI	Cutoff value	Sensitivity (%)	Specificity (%)	PPV (%)	NPV (%)	Accuracy (%)
Serum gastrin concentration (pg/mL)	0.897	0.04	0.819–0.975	355	96.3	74.2	76.5	95.8	84.5
Pepsinogen I (ng/mL)	0.895	0.049	0.813–0.977	9.8	81.5	90.3	87.5	82.4	86.2
Pepsinogen I/II ratio	0.86	0.078	0.765–0.956	1.8	96.3	67.7	71.4	91.3	81.0

*AUROC* area under the receiver operating characteristic curve, *95% CI* 95% confidence interval, *NPV* negative predictive value, *PPV* positive predictive value, *SE* standard error.

## Discussion

In this study, we evaluated the relevance of several clinical findings in AIG diagnosis among (1) patients diagnosed by autoantibody positivity and characteristic histology and (2) non-AIG patients diagnosed based on autoantibody negativity whose *H. pylori* infection status was precisely evaluated. The ROC curve analysis results of the entire study population (N = 332) indicated that the diagnostic performance of advanced endoscopic atrophy (≥ O3 in the Kimura–Takemoto classification) and serology, including PG (PG I, ≤ 20.1 ng/mL; PG I/II ratio, ≤ 1.8) and gastrin (≥ 355 pg/mL), was high; therefore, they have sufficient diagnostic performance for use in daily clinical practice.

As our results showed that advanced endoscopic atrophy itself may be a marker of AIG, it may be useful if highly suspected cases of AIG among patients with advanced atrophy can be further evaluated by serology (i.e., two-stage identification). Based on this idea, an ROC curve analysis of patients with severe gastric atrophy only (≥ O3, N = 58) was performed. Our results indicated that the diagnostic performance of PG (PG I, ≤ 9.8 ng/mL; PG I/II ratio, ≤ 1.8) and gastrin (≥ 355 pg/mL) was also high (AUROC, > 0.85) and that they may be valid predictors. These findings are especially useful when severe atrophy is found during endoscopy, which may be the commonest way of identifying AIG in clinical practice.

In our study, gastrin (355 pg/mL) and PG I/II ratio (1.8) exhibited the same cutoff values for the prediction of AIG in both the overall study cohort and the subset of patients with advanced atrophy. These results highlight how they may be especially useful as serum markers in clinical practice. Considering the high AUROC of these serological markers, we advocate that PG and gastrin should be additionally checked in cases where the diagnosis is challenging, for example, when pathological examination of biopsy specimens does not show findings typical of AIG despite a positive APCA result. Re-biopsy and histological evaluation may be recommended if PG or gastrin levels are strongly suggestive of AIG. Moreover, in serological screening tests, these cutoff values are useful in detecting AIG; in this study; endoscopy was indicated in approximately half (45.2%) of the AIG cases based on a positive serology test using the ABC method.

The endoscopic atrophic grade (Kimura–Takemoto classification) is considered highly objective by several investigators^24,25^, and this atrophy grade was imposed for all upper gastrointestinal endoscopic cases in the Japan Endoscopy Database Project^26^. Although O4 is useful in detecting AIG of high prevalence compared with non-AIG (64.5% vs. 3.0%), new endoscopic criteria are not necessary for AIG diagnosis as it is included as part of grade O3 in the Kimura–Takemoto classification. In AIG diagnosis, endoscopy is often performed merely to take biopsies for histopathological diagnosis with low specificity and objectivity^3,10,27^. However, herein, we demonstrated that the “endoscopic atrophic border” had a Kappa value of 0.654. This is consistent with the report by Terao et al.^17^, wherein high interobserver variability (0.705) was shown in the “degree of atrophy;” however, in the case of “sticky mucosa,” our Kappa value seems to be lower than their value (0.352 vs. 0.604). Although corpus-predominant advanced atrophy compared with the antral region, which we referred to as the “corpus-predominant atrophic pattern,” has been used as a diagnostic criterion^15,16^, it did not have a reliable Kappa value in our study. Regarding the disappearance of the gastric fold in the gastric corpus, it cannot be used as a diagnostic modality because it has a low AUROC value (0.773), even though its Kappa value was > 0.6. Hence, other objective endoscopic findings should be evaluated.

In this study, the cutoff values of gastrin for the diagnosis of AIG obtained from the ROC analysis (355 pg/mL) were higher than those previously reported by Checchi et al.^28^. The cutoff value of gastrin for diagnosing AIG with severe atrophy was reported as 395 pg/mL^11^, and Terao et al.^17^ set hypergastrinemia > 350 pg/mL as one of the criteria for AIG in a multicenter study, which is similar to the 355 pg/mL cutoff value for gastrin in this study. In Checchi et al.’s study, the cutoff value of gastrin (43 pg/mL) was close to normal (< 39.3 pg/mL), suggesting the possible diagnosis of individuals without AIG as having AIG^28^. Simple comparisons with other reports are difficult because of differences in the gastrin unit of measurement (pg/mL and pmol/L)^10,29^.

Regarding PGs, various cutoff values have been reported by several investigators; however, few reports involved an ROC analysis using a histology-based definition of AIG^10,28,30^. The cutoff values for PG I (20.1 ng/mL) and PG I/II ratio (1.8) in our study were similar to those reported by Koc et al.^30^, but significantly lower than those in the studies by Venerito et al.^10^ (PG I, 50 ng/mL; PG I/II ratio, 5) and Checchi et al. (PG I/II ratio, 14)^28^. However, considering that the definition of AIG employed by Venerito et al. was confined to the histology of enterochromaffin-like hyperplasia and the almost normal cutoff value of gastrin reported by Checchi et al., their cutoff values may be set higher than the actual physiological values. The evaluations of the cutoff value of PGs to predict AIG in other studies were inherently limited by various issues. A report evaluated the ROC curve of PGs in appropriately defined patients with AIG, but did not calculate the cutoff value^31^. Three more studies did not analyze the cutoff value in a strictly defined AIG cohort, including participants with multifocal atrophy^32^, pangastritis^29^, or severe atrophy of Operative Link on Gastric Intestinal Metaplasia Assessment > stage 2 and autoantibody positivity^15^. Because Koc et al. only analyzed a limited number of patients (N = 16) with AIG, this is the first report to demonstrate the practical cutoff value of PG in the diagnosis of AIG using a sufficient cohort of strictly defined patients with AIG.

In this study, individuals on acid inhibitors were strictly excluded; however, in clinical practice, the possible effects of acid inhibitory drugs should be considered when using these cutoff values. Hypergastrinemia characterized by gastrin levels over 500 pg/mL, which is induced by PPIs or potassium competitive acid blockers (PCABs)^33,34^, can be a major problem in serological screening for AIG. However, it is unclear whether eradication influences gastrin in patients with AIG because severe hypochlorhydria is induced by AIG in many cases, and eradication may not affect it. As for PG and gastrin, these would theoretically normalize after eradication; however, because of the extremely low PG level and extremely high gastrin level in AIG, eradication treatment does not seem to affect the results of serological screening.

In our study, the median value (IQR) of gastrin was 1,310 (448–2,490) pg/mL, and the PG I level and PG I/II ratio were 6.4 (3.8–15.9) ng/mL and 1.0 (0.6–1.5), respectively, suggesting that the clinical characteristics of AIG in our study were generally comparable to those in previous studies^10,17,27,35^. However, 45.2% of patients with AIG in our study had *H. pylori* infection (19.4% and 25.8% of present and previous infection cases, respectively), an incidence higher than those in previous reports of seropositivity rates (Terao et al.^17^, 7.8%; Venerito et al.^10^, 18.2% [4/22]). In our study, 29/31 patients with AIG (94%) were accurately evaluated by more than two modalities to establish *H. pylori* infection, and eradication was diagnosed by normalization of the urea breath test, which may explain the high infection rate in our study.

The prevalence of AIG (5.8%, 31/536) in this study is higher than the reported overall prevalence in Japan (0.49%)^16^. Eighteen patients with AIG, who were included in the analysis, were already being followed-up at our hospital with regular endoscopy. Thus, the prevalence of first-visit cases was 13/536 (2.4%), which is still higher than the national prevalence rate. However, we believe that this is reasonable given how rare cases tend to cluster at major referral hospitals, such as ours.

This study has some limitations. First, the identification of patients with AIG was based only on autoantibodies and histological findings. In this study, 2/31 histologically defined AIG cases (6.4%) had normal gastrin levels, suggesting that false positive cases were inevitably included (although there is a possibility that these were very early stage AIG cases), and that additional criteria using serological markers, such as gastrin and/or PG, may be required to increase the accuracy of the diagnosis of AIG, as described earlier. Second, other endoscopic findings, including remnant oxyntic mucosa, scattered minute whitish protrusions, and intestinal metaplasia were not evaluated. They may be potential predictive factors of AIG and should, therefore, be evaluated. Furthermore, the small sample size of patients from a single institution may limit the generalizability of the obtained results.

In conclusion, in this study, we demonstrated that endoscopic findings of severe atrophy (≥ O3 in the Kimura–Takemoto classification) are useful predictors of AIG. High serum gastrin levels and low PG I and I/II ratio are also predictors of AIG. The cutoff values of serum gastrin levels (≥ 355 pg/mL) and low PG I/II ratio (≤ 1.8) can be used in both the general population and those with endoscopic atrophy (≥ O3). Thus, patients with advanced atrophy with a strong suspicion of AIG can be further evaluated using these cutoff values. Clinicians should also note that AIG can be suspected by serology, especially when false negative or positive results are suspected or during serological screening for gastritis involving PG and/or gastrin.

## Methods

### Ethics approval

All procedures performed in studies involving human participants were in accordance with the ethical standards of the institutional and/or national research committee and with the 1964 Declaration of Helsinki and its later amendments or comparable ethical standards. The study design was approved by the Ethics Committee of Tokyo Dental College Ichikawa General Hospital (approval numbers: I-283 RII/2016, I-283 RIII/2017, I-283 RIV/2018, I-283 RV/2019, and I-283 RVI/2021). Written informed consent was obtained from all participants.

### Study design and participants

In this cross-sectional study, 536 consecutive patients who underwent upper gastrointestinal endoscopy at Tokyo Dental College Ichikawa General Hospital between January 2017 and March 2020 were enrolled after obtaining written informed consent. The exclusion criteria for this study were as follows: (1) use of histamine-2 receptor antagonists, PPIs, or PCABs within the preceding 2 months; (2) presence of viral diseases; (3) pregnancy or lactation; (4) presence of renal and/or liver dysfunction; or (5) a past history of gastric cancer or any type of esophageal or gastric surgery^36^. Most of the excluded individuals were excluded by interview before enrollment.

A flowchart of the target group selection is shown in Fig. 1. “AIG” was defined based on both (1) seropositivity for APCA and/or AIFA, and (2) histological findings consistent with AIG. Patients positive for either APCA or AIFA were classified into the following two subgroups: patients in whom biopsy of the gastric body was performed, and those without biopsy. The latter were excluded from the analysis. For AIG diagnosis, in the case of APCA positivity only, at least three of the five histological findings compatible with AIG, as described in the “Histology” section, were necessary. In the case of AIFA positivity, irrespective of APCA, at least two of the five histological findings were necessary because the sensitivity of AIFA in diagnosing AIG is considerably higher than that of APCA^8^. Non-AIG controls were defined as individuals who tested negative for APCA, and 57 were excluded due to inadequate APCA or gastrin data, 13 due to PPI treatment, and 3 due to history of gastric cancer.

*H. pylori* infection status in the control participants was defined as follows: Patients who were seronegative for APCA and with a clear history of successful eradication, confirmed by a negative result on either a urea breath or stool antigen test, were defined as “*H. pylori-*eradicated patients.” In contrast, those with a result < 2.5% in the urea breath test were classified as negative according to the manufacturer’s recommended cutoff value. Patients negative for APCA and with a history of eradication were further classified into two subgroups: patients positive (≥ 10 U/mL) and negative (< 10 U/ml) for *H. pylori* antibody titer. The former was defined as “present *H. pylori* infection cases.” The latter individuals with an atrophy grade from C0 to C1, which is regarded as an atrophy score of 0 (negative) in the Kyoto classification^37^, were defined as “*H. pylori*-uninfected cases.” The patient group negative for *H. pylori* antibody (< 10 U/mL) and endoscopic atrophy (≥ C-2) may include those who are *H. pylori*-infected and those with previous infection-induced atrophic gastritis^36,38,39^. Among these patients, those with at least one positive urea breath, stool antigen, or culture test were classified as “present *H. pylori* infection cases,” whereas previous infection-induced atrophic gastritis cases were defined when negative results were obtained in urea breath or stool antigen tests, as we reported previously^40^. If a urea breath or stool antigen test was not performed, individuals with an eradication history and those with endoscopic atrophy (≥ C2) and seronegative results of *H. pylori* were excluded.

*H. pylori* infection status was defined differently in patients with AIG because of the extreme case bias. Those with at least one positive urea breath, stool antigen, *H. pylori* antibody titer, or culture test were classified as “present *H. pylori* infection cases”. The cutoff value for a positive diagnosis in the urea breath test was set at 5% instead of 2.5%^41^ because false-positive results tend to be obtained in AIG. Patients with an apparent history of successful eradication, confirmed by a negative result in either a urea breath or stool antigen test, were defined as “*H. pylori-*eradicated patients.” The cutoff value for a negative result in these urea breath tests was strictly 2.5%. It is difficult to differentiate between *H. pylori-*uninfected individuals and those with a previous infection among patients with AIG; thus, for simplicity, patients other than those with a present infection or history of eradication were defined as *H. pylori-*uninfected.

To evaluate the diagnostic validity and determine the threshold values of the serological tests and endoscopic findings for predicting AIG, an ROC curve analysis was performed.

### Measurement of serum biomarkers

Fasting serum samples (after an overnight fast) were collected before endoscopy. PG, gastrin, *H. pylori* antibody titers, and APCA were examined in the enrolled patients. Briefly, serum PG I and PG II levels were measured using a commercial chemiluminescence enzyme immunoassay kit (Architect Pepsinogen I, Pepsinogen II, Abbott Japan Co. Ltd., Tokyo, Japan); *H. pylori* IgG antibodies were measured using an enzyme immunoassay kit (E Plate “Eiken” *H. pylori* antibody, Eiken Chemical Co. Ltd., Tokyo, Japan); and gastrin level was determined with radioimmunoassay (Gastrin RIA Kit II, Fujirebio Diagnostics Co., Ltd., Tokyo, Japan), as previously reported^36^. In patients clinically suspected of having AIG (e.g., severe atrophy of ≥ O3 according to the Kimura–Takemoto classification or APCA positivity), thyroglobulin antibodies, thyroid microsomal antibodies, serum vitamin B12, and AIFA were additionally measured. APCA and AIFA measurements were outsourced to SRL Co. Ltd. (Tokyo, Japan), and thyroglobulin and thyroid microsomal antibody measurements were performed by LSI Medience Co., Ltd. (Tokyo, Japan). Anemia was defined as hemoglobin < 13 g/dL (men) or 11.4 g/dL (women); vitamin B12 level < 233 pg/mL was considered low; and pernicious anemia was defined as vitamin B12 < 233 pg/mL, mean corpuscular volume (MCV) > 80 fL, and hemoglobin < 13 g/dL (men) or 11.4 g/dL (women)^17^. Hashimoto’s disease was defined as positivity for thyroglobulin antibodies or thyroid microsomal antibodies. APCA and AIFA were analyzed using a fluorescent antibody test and chemiluminescence enzyme immunoassay, respectively. APCA was considered positive when fluorescence was produced at a dilution of tenfold or more according to the manufacturer’s instruction. Although there is limited information on the sensitivity of APCA, a study reported that 33.59% of APCA-positive cases were diagnosed as AIG^7^.

### Histology

**Figure 3:**
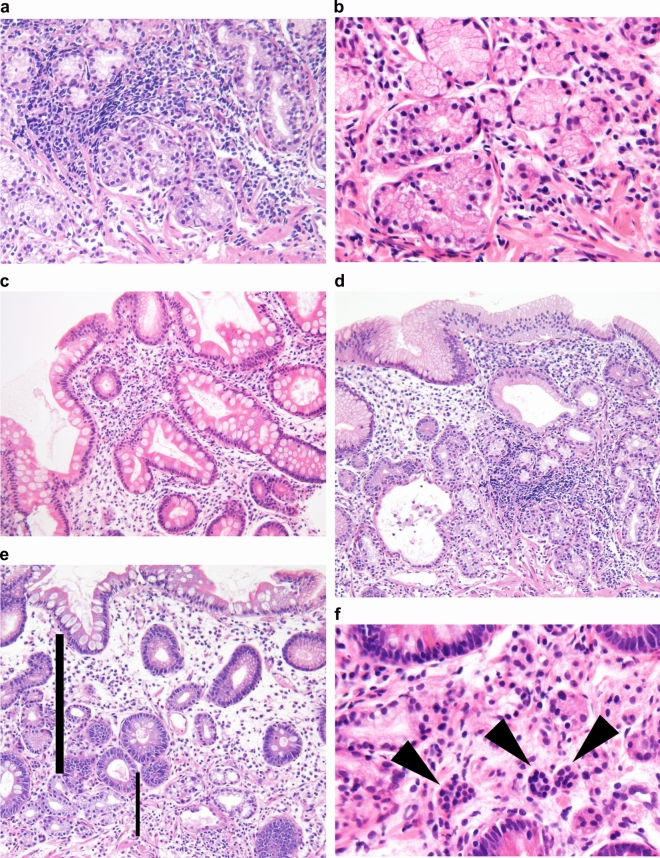
Targeted histological findings of autoimmune gastritis. Oxyntic mucosal atrophy with lymphocytic infiltrates (**a**). Epithelium of pseudo-pyloric (**b**) and intestinal metaplasia (**c**). Diffuse lymphocyte cell infiltration, which is heavier in the deep portion than in the lamina propria (**d**). Increased proportion of gastric pits (bold line) induced by severe atrophy, which was defined as the proportion of gastric pits (bold line)/gastric duct (thin line) (**e**). Enterochromaffin-like cell hyperplasia (arrowhead) (**f**).

Patients positive for either APCA or AIFA with available gastric corpus biopsies were histologically evaluated by an experienced pathologist (AS) who was blinded to the patients’ endoscopic and serological information and medical charts. Biopsy specimens were fixed with buffered formalin and stained using hematoxylin and eosin. The following five findings were evaluated to diagnose AIG based on previous reports^3,9–14^: (1) oxyntic mucosal atrophy, (2) pseudo-pyloric and intestinal metaplasia, (3) diffuse lymphocytic cell infiltration, (4) proportion of gastric pits/duct in the gastric mucosa (> 1.0 or none), and (5) enterochromaffin-like cell hyperplasia. Typical histological findings are shown in Fig. 3a–f.

### Endoscopic examination

**Figure 4:**
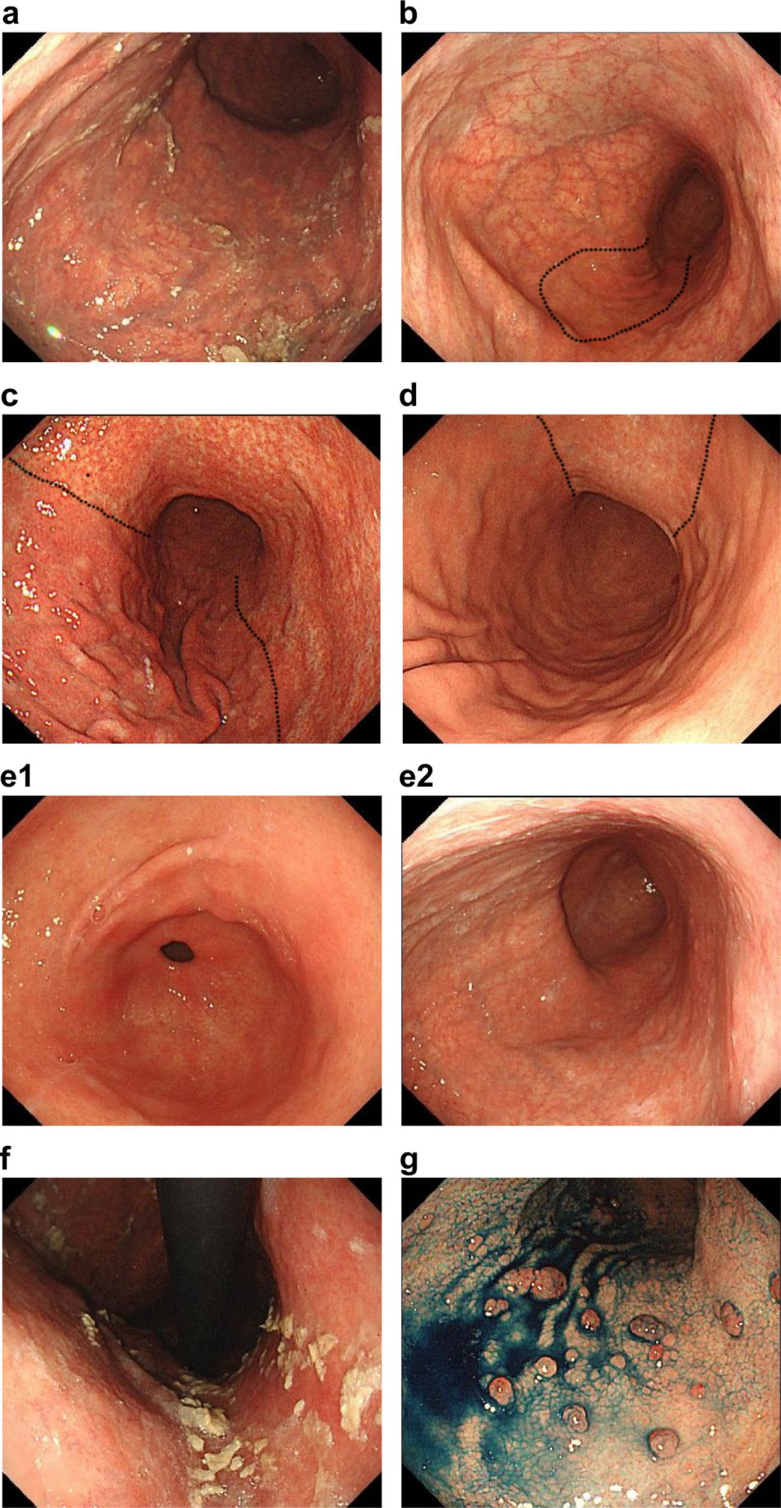
Representative endoscopic findings associated with autoimmune gastritis. Endoscopic findings of O4 with vascular visibility in the entire area of the greater curvature of the corpus (**a**). O3 with spared vascular visibility in a part of the greater curvature of the corpus (**b**). O2 with spared vascular visibility in the entire area of the greater curvature of the corpus (**c**). O1 with vascular visibility in the lesser curvature of the corpus (**d**). The endoscopic atrophic border is indicated by a dotted line (**b**–**d**). Endoscopic findings of corpus-predominant advanced atrophy (O4) with a non-atrophic antral part (antrum [**e1**] and corpus [**e2**]). Endoscopic findigs of the disappearance of the fold in the greater curvature of the gastric body (**a**, **b**, **e2**). Typical endoscopic findings of sticky adherent dense mucous (**f**) and multiple hyperplastic polyps in the proximal stomach (**g**).

Upper gastrointestinal endoscopy was performed using an Olympus Elite system and Olympus electronic panendoscopes, including the GIF-HQ290 or GIF-HQ290Z (Olympus, Optical Co. Ltd, Tokyo, Japan). Based on previous reports^13,14,16,17^, the following five endoscopic findings considered specific for AIG were evaluated individually: (1) endoscopic atrophic grade, (2) characteristic corpus-predominant atrophic pattern (corpus-predominant advanced atrophy compared with the antral part), (3) disappearance of the fold in the greater curvature of the gastric body in the sufflation state, (4) presence of sticky adherent dense mucous, and (5) hyperplastic polyps. Sticky adherent dense mucous has a denser, creamy white-yellowish color and firmly adheres to the mucosa, as defined by Terao et al*.*^17^. The endoscopic atrophic grade was defined according to the Kimura–Takemoto classification^42^, which categorized gastric mucosal atrophy into closed (C1–3) and open types (O1–3). We added “O4” for severe endoscopic atrophic corpus gastritis, which was defined as marked vascular visibility observed not only on the lesser curvature but also on the whole greater curvature of the corpus. This is included in the O3 category of the Kimura–Takemoto classification and is similar to the definition provided by Terao et al.^17^; however, it was not associated with the disappearance of the fold according to our definition. Figures 4a–d depict typical endoscopic findings of O4 (Fig. 4a), O3 (Fig. 4b), O2 (Fig. 4c), and O1 (Fig. 4d). Figure 4e shows the endoscopic findings of the corpus-predominant atrophic and non-atrophic patterns in the antrum (Fig. 4e-1), despite severe corpus atrophy (Fig. 4e-2). Figures 4a, 4b, and 4e-2 show the disappearance of the fold. Figure 4f shows sticky adherent dense mucous, and Fig. 4g shows multiple hyperplastic polyps.

Three experienced endoscopists (KN, YH, and HH), blinded to the clinical, serological, and histological status of the patients, independently evaluated the five endoscopic images and determined the final endoscopic findings by consensus.

### Statistical analyses

Serum gastrin and PG values are presented as medians (IQR), and statistical analyses were performed using nonparametric tests. The Mann–Whitney *U* test was used to determine the significance of the differences between patients with and without AIG. The χ^2^ test or Fisher’s exact test was performed to analyze categorical variables. Interobserver agreement between the diagnosis of endoscopic findings was analyzed using Kappa values. The strength of agreement was determined based on the guidelines by Landis and Koch^43^, and ≥ 0.6 was considered suitable. AUROC values were used to identify the optimal cutoff value. Comparison of AUROC values was performed using the Delong test. Sensitivities, specificities, positive predictive values, negative predictive values and overall accuracy were also calculated. All statistical analyses were performed using SPSS (version 25; SPSS, Chicago, IL, USA) and EZR (Saitama Medical Center, Jichi Medical University, Saitama, Japan)^44^, and a two-sided *P* < 0.05 was considered statistically significant.

## Supplementary Information

Supplementary Tables.

## Data Availability

The datasets generated during and/or analyzed during the current study are not publicly available due to confidentiality but are available from the corresponding author on reasonable request.

## Abbreviations

AIG: Autoimmune gastritis
AIF: Anti-intrinsic factor
APCA: Anti-parietal cell antibody
AUROC: Areas under the receiver-operating characteristic curve
CIs: Confidence intervals
IQR: Interquartile range
NPV: Negative predictive value
OR: Odds ratio
PCAB: Potassium competitive acid blocker
PG: Pepsinogen
PPI: Proton pump inhibitor
PPV: Positive predictive value
ROC: Receiver-operating characteristic
SE: Standard error
