# Supplementary material for: Relevance of pepsinogen, gastrin, and endoscopic atrophy in the diagnosis of autoimmune gastritis
Source: Sci Rep. 2022 Mar 10;12:4202. doi: 10.1038/s41598-022-07947-1 (PMC8913737; doi:10.1038/s41598-022-07947-1)
Supplement: Supplementary file 1 — Supplementary Tables. [file 41598_2022_7947_MOESM1_ESM.pdf]

Relevance of Pepsinogen, Gastrin, and Endoscopic Atrophy in the Diagnosis of Autoimmune Gastritis

Hiroshi Kishikawa, Kenji Nakamura, Keisuke Ojiro, Tadashi Katayama, Kyoko Arahata, Sakiko Takarabe, Aya Sasaki, Soichiro Miura, Yukie Hayashi, Hitomi Hoshi, Takanori Kanai, and Jiro Nishida

Supplementary Table S1. Histological findings of autoimmune gastritis

Histological findings	N (%)
Oxyntic mucosal atrophy	31/31 (100%)
Pseudo-pyloric or intestinal metaplasia	28/31 (90.3%)
Pseudo-pyloric metaplasia	19/31 (61.3%)
Intestinal metaplasia	25/31 (80.6%)
Decreased proportion of gastric pits/gastric duct	27/31 (87.1%)
Diffuse lymphocyte cell infiltration	31/31 (100%)
Enterochromaffin-like cell hyperplasia	6/31 (19.4%)

Supplementary Table S2. Interobserver variability in endoscopic diagnosis suggestive of autoimmune gastritis among the three endoscopists

**Endoscopic findings**	**Kappa value (95% CI)**			**Mean ± SD**
	**KN to YH**	**KN to HH**	**YH to HH**	
Endoscopic atrophic grade*	0.698 (0.657-0.740)	0.643 (0.595-0.691)	0.621 (0.574-0.667)	0.654±0.04
Disappearance of the gastric fold	0.684 (0.558-0.811)	0.539 (0.409-0.669)	0.642 (0.522-0.763)	0.622±0.07
Corpus predominant advanced atrophy in comparison to the antral part	0.309 (0.149-0.470)	0.255 (0.076-0.435)	0.441 (0.272-0.610)	0.335±0.1
Sticky mucosa	0.235 (0.119-0.351)	0.240 (0.108-0.372)	0.580 (0.470-0.691)	0.352±0.2
Hyperplastic polyp	0.582 (0.369-0.795)	0.505 (0.250-0.759)	0.461 (0.231-0.690)	0.516±0.06

CI, confidence interval; SD, standard deviation.

* Kappa value was regarded as “moderate (0.6–0.8)” based on the Landis and Koch scale and was used as an analyzed item.

Supplementary Table S3. Baseline characteristics of patients with severe endoscopic atrophy (≥O3 on the Kimura–Takemoto classification)

Baseline characteristics	Autoimmune gastritis (N=27)	Non-autoimmune gastritis (N=31)	P-value*
Male, N (%)	15 (55.6%)	18 (58.1%)	0.847
Age, years, median (IQR)	73 (68–76)	75 (70–76)	0.419
Pepsinogen I (ng/mL), median (IQR)	5.9 (3.7–9.4)	18.9 (15.1–32.7)	<0.001*
Pepsinogen II (ng/mL), median (IQR)	7.4 (4.9–9.2)	6.1 (4.6–14.4)	0.806
Pepsinogen I/II ratio, median (IQR)	0.9 (0.6–1.2)	3.3 (1.2–4.3)	<0.001*
H. pylori antibody titer ≥10 U/ml, N (%)	2/27 (7.4%)	17/31 (54.8%)	<0.001*
H. pylori infection status			
uninfected/present infection/ eradicated/past infection	17/4/6/0 (63.0%/14.8/22.2%/0%)	0/17/11/3 (0%/54.8%/35.5%/9.7%)	<0.001*
Gastrin (pg/mL), median (IQR)	1500 (637–2830)	196 (108–561)	<0.001*
<100/100–350/350–1,000/>1,000 pg/mL	0/1/8/18 (0%/3.7%/29.6%/66.7%)	7/16/5/3 (22.6%/51.6%/16.1%/9.7%)	<0.001*
Anti-parietal cell antibody positive, N (%)	25/27 (92.6%)	0/31 (0%)	<0.001*
Anti-intrinsic factor antibody positive, N (%)	15/25 (60%)	NA	NA
Endoscopic atrophic border†, N (%)			
O3/O4	7/20 (25.9%/74.1%)	22/9 (71.0%/29.0%)	<0.01*
Disappearance of the gastric fold, N (%)	19/27 (70.4%)	16/31 (51.6%)	0.145

Data are provided as numbers (%) or median (interquartile range [IQR]).
* *P*-value: Fisher's exact test or Man–Whitney *U* test; autoimmune gastritis vs. non-autoimmune gastritis.
